# Assessment of Cortical Dysfunction in Patients with Intermittent Exotropia: An fMRI Study

**DOI:** 10.1371/journal.pone.0160806

**Published:** 2016-08-08

**Authors:** Qian Li, Junxing Bai, Junran Zhang, Qiyong Gong, Longqian Liu

**Affiliations:** 1 Department of Optometry and Visual Science, West China School of Medicine, Sichuan University, Chengdu, Sichuan Province, P.R. China; 2 Department of Ophthalmology, MEM Eye Care System, Beijing, P.R. China; 3 Huaxi MR Research Center, Department of Radiology, West China Hospital, Sichuan University, Chengdu, Sichuan Province, P.R. China; Shenzhen Institutes of Advanced Technology, CHINA

## Abstract

Neural imaging studies have found the connection between strabismus and brain cortex. However, the pathological mechanisms of intermittent exotropia are still not fully understood. In the present study, changes of binocular fusion related cortices in intermittent exotropia were investigated with blood oxygen level dependent functional magnetic resonance imaging. Activated cortices induced by fusion stimulus were found to be distributed in several regions such as bilateral middle occipital gyrus, bilateral middle temporal gyrus, left superior parietal lobule and so on. Compared with normal subjects, the increased activation intensity was observed in bilateral superior parietal lobule and inferior parietal lobule in subjects with intermittent exotropia. These findings indicate that binocular fusion involves a complicated brain network including several regions. And cortical activities of bilateral superior parietal lobule and inferior parietal lobule compensate for the binocular fusion dysfunction in intermittent exotropia.

## Introduction

Intermittent exotropia is a common type of concomitant exotropia, with the incidence ranging from 0.12% to 3.9% in different countries and ethnic groups [1–3]. It is also a state between exophoria and constant exotropia, and patients with intermittent exotropia have the ability to control the exodeviation with fusion mechanisms [4]. The pathological mechanisms of intermittent exotropia might be associated with multiple factors. Some studies investigated the abnormalities of ocular muscles. Altered ultrastructures of extraocular muscles like atrophy and fibrosis were observed with light and electron microscopy[5]. Another studies concentrated their attention on binocular vision. The defective binocular fusion was more considered as the possible cause of intermittent exotropia [6–8].

Binocular fusion is the ability of the brain to synthesize images from both two retinas, which is a complex cerebral activity including motor and sensory fusion [9]. It is formed gradually since 5 months after birth in human, along with the development of visual system [10]. Completely developed binocular fusion is important for ocular alignment. Any abnormal visual experience such as anisometropia, ametropia, and form deprivation may impair the binocular fusion through its influences on the development of binocular neurons [11]. Abnormalities of binocular fusion will not keep the coordination or balance between two visual axes, thus lead to unstable eye alignment, which becomes one of the possible causes of strabismus.

Previous studies about intermittent exotropia mainly concentrated on its clinical characteristics and the outcomes of operation [12–13]. Neural mechanisms are rarely mentioned. Neural imaging techniques such as positron emission computerized tomography (PET) and magnetic resonance imaging (MRI) make it possible for us to explore the mechanisms of human strabismus. Connections between brain cortex and strabismus have been reported in some studies [9,14–15]. It has been reported increased activated areas in the left cingulate gyrus, bilateral precuneus and left angular gyrus, which may compensate for the fusion dysfunction in infantile esotropia [9]. For patients with concomitant exotropia, plastic changes of brain were observed with voxel-based morphometry (VBM). Smaller volumes of gray matter were found in occipital eye field and parietal eye field, and greater volumes in frontal eye field, supplementary eye field and prefrontal cortex [15]. Characteristics of strabismus may vary greatly in different types. As a special type of concomitant exotropia, the recognition of neural mechanisms in intermittent exotropia may help us to have a deep understanding about strabismus and provide theory evidences for treatment.

In the present study, a series of pictures with visual disparities were used to produce binocular fusion visual stimulus. We explored the binocular fusion related cortices in intermittent exotropia and normal subjects with blood oxygenation level dependent-functional magnetic resonance imaging (BOLD-fMRI). It will be helpful for us to further understand the binocular fusion of intermittent exotropia and the neural mechanisms of strabismus.

## Methods

### Subjects

In this cross-sectional study, nine intermittent exotropia subjects and eight control subjects were recruited in West China Hospital of Sichuan University from July 2015 to February 2016. All participants underwent a series of ophthalmologic examinations including a slit-lamp examination, fundus examination, test of ocular motility, Titmus Stereo Test (Stereo Optical Inc, Chicago, IL, USA), retinoscopy refraction and measurement of best corrected visual acuity. Angles of deviation were quantified with a prism and alternate cover test. All subjects were right-hand and had no history of strabismus surgery before the beginning of the present study, as well as amblyopia and other ocular diseases, psychiatric disorders or brain abnormalities.

This study followed the tenets of Declaration of Helsinki and was approved by the Ethics Committee of West China Hospital, Sichuan University. Written informed consents were obtained from all subjects after we explained the research and its possible complications verbally and in written, and the information of patients was anonymized and identified prior to analysis.

### Visual stimuli

A block design paradigm was used in this study. The visual stimuli were displayed and projected on a translucent screen which could be seen by the subject through an angled mirror positioned above the subjects’ eyes. The distance between translucent screen and subjects’ eyes was about 100 centimeters and the visual angle was 17°. Both two eyes were corrected to best-corrected visual acuity with lenses during the scanning. Besides that, they also need to add red-blue glasses.

Each block consisted of 6 pictures as follows: three “stimulus” pictures including rabbit pictures with static visual disparities, rabbit pictures with dynamic visual disparities (it can be observed the movement of limbs in rabbit) and rabbit pictures without visual disparity (control stimulus). Each stimulus picture displayed randomly for 16 seconds, and inserted with three “break” pictures (white cross in the center with black background) displaying for 14 seconds. Each block lasted for 90 seconds and was repeated 5 times for a total of 450 seconds.

### Image Acquisition

MRI scannings were performed in a 3.0T MR scanner system with a standard eight-channel head coil (EXCITE, GE Signa, Milwaukee, USA). During the scanning, foam pads were filled around head and earplugs for all subjects to minimize head motion and scanner noise, respectively.

Three dimensional T1 weighted anatomical images were collected with the following scan parameters: repetition time (TR) = 8.5ms, echo time (TE) = 3.4ms, flip angle = 12°, slice thickness = 1mm. Functional images were obtained with an echo planar imaging (EPI) sequence, and the scan parameters were as follows: TR = 2000ms, TE = 30ms, flip angle = 90°, matrix = 64×64, field of view = 240×240mm^2^, slice thickness = 5mm.

### Data analysis

The statistical parametric mapping package (SPM8, http://www.fil.ion.ucl.ac.uk/spm) running under MATLAB 7.0 (The MathWorks, Natick, USA) was used for image preprocessing. The first twenty volumes were discarded to ensure stable magnetization and subjects’ adaption to the circumstances. Slice timing and realignment for head motion correction were then performed for the remaining 200 images. Data of one intermittent exotropia subject and one control subject were excluded from following analysis because either translation or rotation of their head motion exceeded 1mm or 1° on any axis.

For fMRI data, after preprocessing in SPM8, data from subjects in the same group were combined and statistically analyzed to obtain the averaged images of activation. The paired t-test was applied to compare the differences of activated areas induced by fusion stimulus and control stimulus in the same group. And the group t-test was used to compare the differences induced by stimulus. A false discovery rate (FDR) was set at a level of p<0.001, uncorrected, and the extent threshold was set with 10 voxels, which means significant activated areas were defined as the number of continuous activated voxels reached more than 10 voxels. We recorded the volume and intensity of activation, and the data were then spatially normalized to stereotaxic space and superimposed onto the respective averaged anatomic images.

For demographic comparisons, the statistical analysis was performed with SPSS Statistics (SPSS Inc, Version 18.0, Chicago, USA). Two-sample two-tailed t-test was performed for age comparison and Fisher’s exact probability test for gender. Significant difference was set at a level of p<0.05.

## Results

### Demographic comparisons

Demographic and clinical information of 8 intermittent exotropia subjects (3 males, 5 females; age: 23.8±6.8 years) were displayed in Table 1. In addition, 7 normal control subjects (3 males, 4 females; age: 25.0±2.1 years) were well matched in age (two-sample two-tailed t-test, t = -0.443, p = 0.669) and gender (Fisher’s exact probability test, p>0.99).

**Table 1:** Demographic and clinical datum in intermittent exotropia subjects.

Subject	Gender	Age (years)	Refractive status	Visual acuity (logMAR)	Angle of deviation (^△^)	
					Near (33cm)	Distant (6m)
**Sub01**	F	18	**OD** -5.00	-0.1	-25	-30
			**OS** -4.25	-0.1		
**Sub02**	M	18	**OD** PL	0	-40	-35
			**OS** PL	0		
**Sub03**	M	22	**OD** -4.25/-0.50×20	-0.1	-60	-60
			**OS** -3.00/-0.75×5	-0.1		
**Sub04**	F	20	**OD** -0.75	0	-55	-55
			**OS** +1.00	0		
**Sub05**	F	37	**OD** -4.50/-1.50×25	0	-90	-80
			**OS** -2.50/-1.00×150	0		
**Sub06**	F	25	**OD** -4.00	0	-40	-30
			**OS** -4.50/-0.50×130	0		
**Sub07**	F	20	**OD** PL	-0.2	-75	-75
			**OS** PL	0		
**Sub08**	M	31	**OD** -1.50	0	-50	-50
			**OS** -1.25	0		

### Activated brain regions in normal subjects

**Fig 1:**
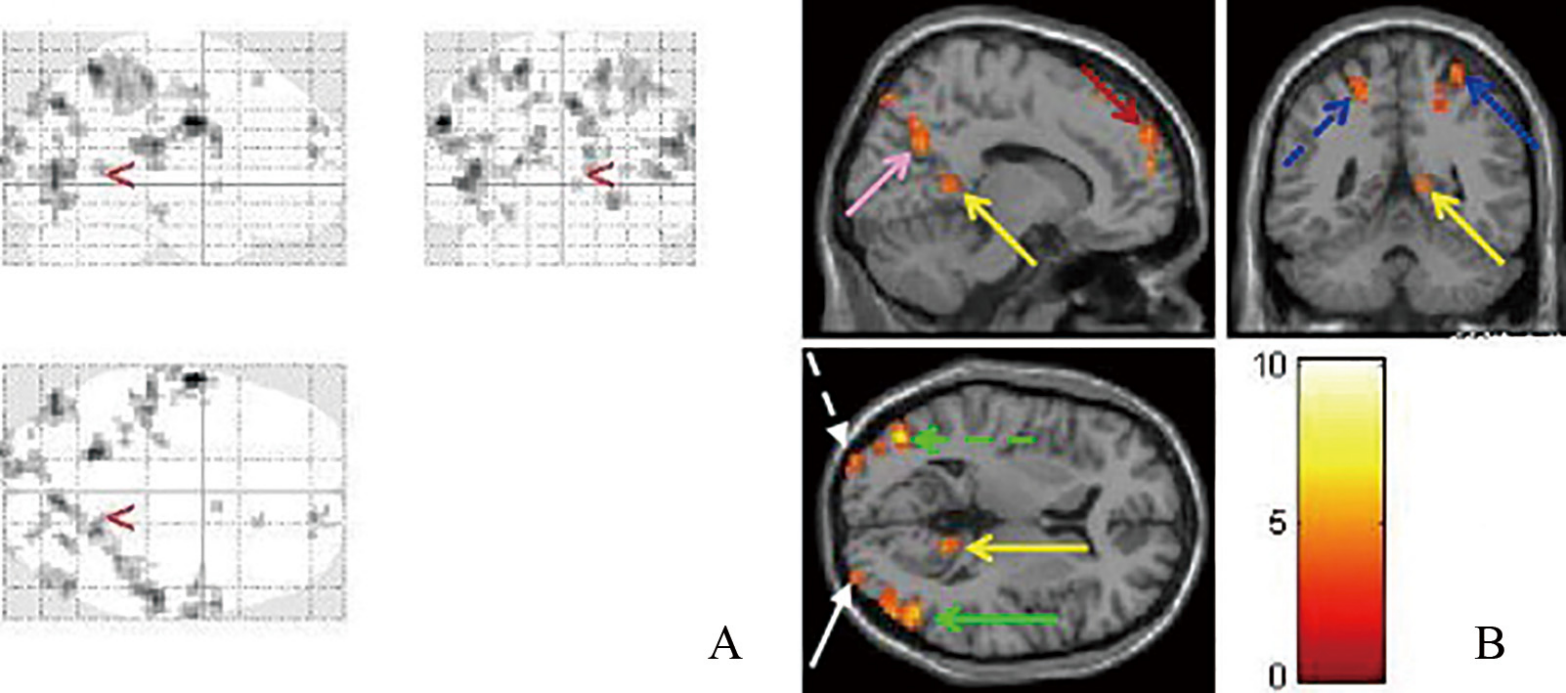
Brain activities images of normal subjects with the binocular fusion stimulus. (A) Glass brain shows activated areas. (B) Activation areas are showed in sagittal, coronal and horizon planes. The yellow, pink, blue, red, green and white arrows point to the posterior cingulated gyrus, precuneus, postcentral gyrus, superior frontal gyrus, middle temporal gyrus and middle occipital gyrus, respectively.

Cortical activation of normal subjects were found to be distributed in following regions: occipital lobe (bilateral middle occipital gyrus, right fusiform gyrus, left cuneus), temporal lobe (bilateral middle temporal gyrus), parietal lobe (bilateral postcentral gyrus, bilateral inferior parietal lobule, bilateral precuneus, left superior parietal lobule), frontal lobe (bilateral precentral gyrus, right superior frontal gyrus, right middle frontal gyrus, right inferior frontal gyrus), and other regions including right posterior cingulate and caudate nucleus (Table 2 and Fig 1).

**Table 2:** Brain activitation in normal subjects with the binocular fusion stimulus.

Cluster	Voxel number	Activated brain regions	T values	Talairach coordinates		
				X	Y	Z
1	80	Left middle occipital gyrus	6.62	-21	-102	12
		Left cuneus	5.79	-12	-93	21
2	67	Right middle occipital gyrus	6.28	51	-69	6
		Right middle temporal gyrus	5.16	48	-78	9
		Left superior parietal lobule	7.96	-21	-54	60
3	144	Left middle temporal gyrus	7.18	-42	-75	9
4	12	Left postcentral gyrus	4.09	-60	-24	42
		Right postcentral gyrus	3.67	54	-27	51
5	56	Left inferior parietal lobule	6.06	-54	-27	21
6	188	Right inferior parietal lobule	5.14	42	-42	57
		Right inferior parietal lobule	5.61	36	-42	51
7	31	Left precuneus	4.72	-18	-84	42
8	75	Right precuneus	5.16	12	-63	30
		Right precuneus	7.51	6	-72	42
9	92	Left precentral gyrus	4.37	-54	-9	51
10	64	Right precentral gyrus	6.19	66	-3	30
		Right inferior frontal gyrus	5.81	60	6	24
11	34	Right fusiform gyrus	5.73	27	-69	-9
12	10	Right superior frontal gyrus	4.16	15	30	57
		Right caudate nucleus	4.14	9	9	0
13	24	Right superior frontal gyrus	5.36	15	57	33
		Right superior frontal gyrus	4.22	9	63	30
14	21	Right middle frontal gyrus	3.71	42	0	51
15	17	Right posterior cingulate	5.02	15	-51	9

p<0.001 (FDR uncorrected), voxels>10

### Activated brain regions in subjects with intermittent exotropia

**Fig 2:**
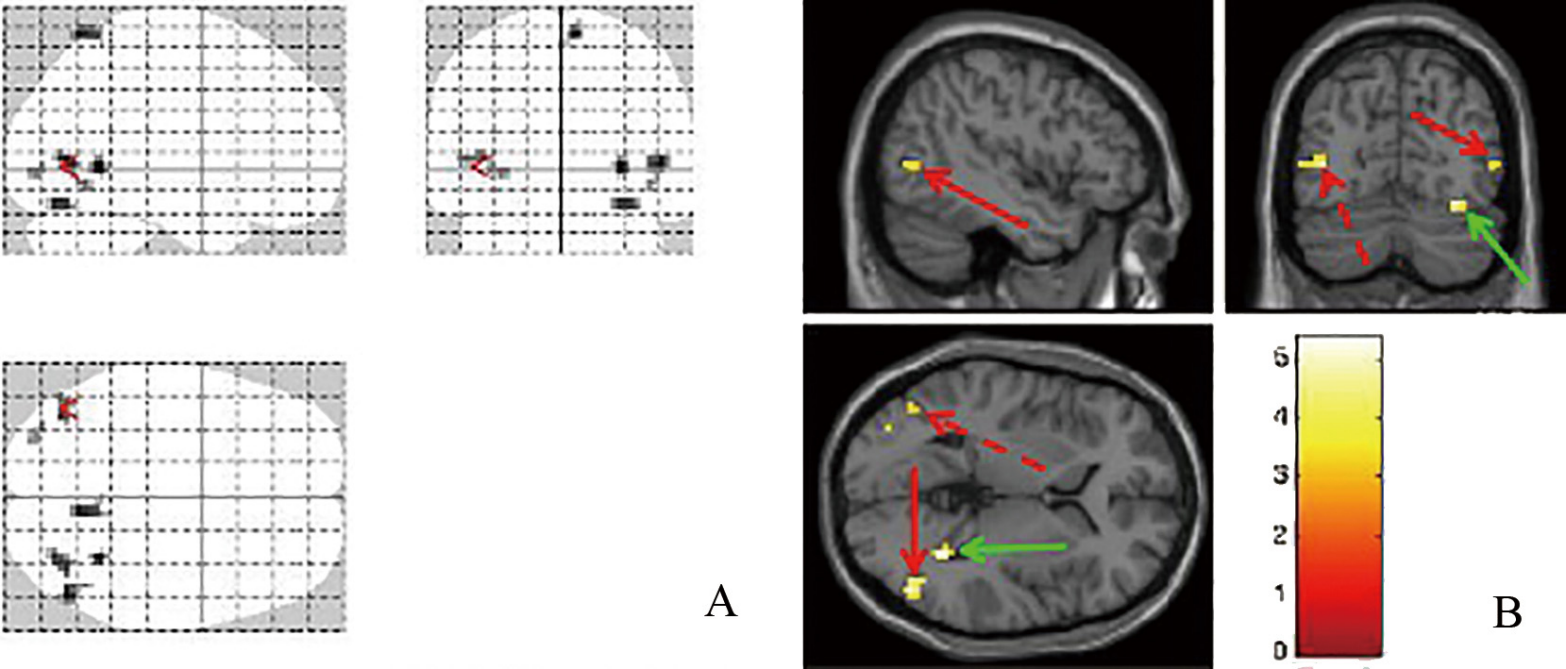
Brain activities images of intermittent exotropia subjects with the binocular fusion stimulus. (A) Glass brain shows activated areas. (B) Activation areas are showed in sagittal, coronal and horizon planes. The red and green arrows point to the middle occipital gyrus and fusiform gyrus, respectively.

In intermittent exotropia subjects, cortical activation mainly distributed in following regions: bilateral middle occipital gyrus, right inferior temporal lobule, right precuneus, fusiform gyrus and lingual gyrus. The sites and degree of activation were showed in Table 3 and Fig 2.

**Table 3:** Brain activitation in intermittent exotropia subjects with the binocular fusion stimulus.

Cluster	Voxel number	Activated brain regions	T values	Talairach coordinates		
				X	Y	Z
1	18	Left middle occipital gyrus	4.67	-39	-72	6
		Left middle occipital gyrus	4.11	-48	-69	6
2	11	Left middle occipital gyrus	3.71	-30	-84	0
3	31	Right middle occipital gyrus	5.03	51	-69	3
		Right inferior temporal lobule	3.94	45	-60	-6
4	34	Right precuneus	4.90	6	-63	69
		Right precuneus	3.26	3	-51	63
		Right precuneus	3.19	9	-48	75
5	23	Right fusiform gyrus	4.48	36	-69	-18
		Right lingual gyrus	4.62	30	-75	-15

p<0.001 (FDR uncorrected), voxels>10

### Comparisons of activated areas between normal and intermittent exotropia subjects

**Fig 3:**
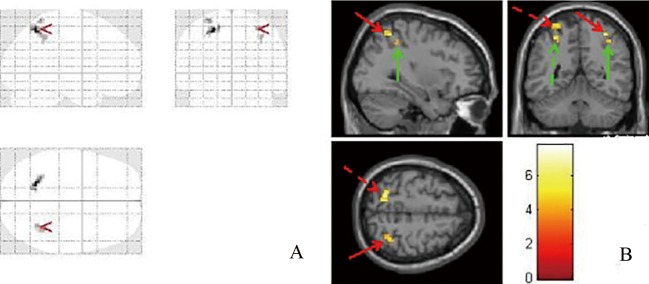
Brain images of increased activation in intermittent exotropia subjects compared with controls. (A) Glass brain shows activated areas. (B) Activation areas are showed in sagittal, coronal and horizon planes. The red and green arrows point to the superior parietal lobule and inferior parietal lobule, respectively.

Compared with normal subjects, increased activation intensity of bilateral superior parietal lobule and inferior parietal lobule were observed in intermittent exotropia subjects (Table 4, Fig 3).

**Table 4:** Increased brain activitation in intermittent exotropia subjects compared with controls under the condition of binocular fusion stimulus.

Cluster	Voxel number	Activated brain regions	T values	Talairach coordinates		
				X	Y	Z
1	44	Left inferior parietal lobule	5.66	-30	-51	51
		Left superior parietal lobule	7.75	-21	-57	57
2	12	Left superior parietal lobule	5.34	-30	-51	69
3	13	Right superior parietal lobule	5.16	33	-51	57
4	15	Right inferior parietal lobule	4.89	36	-45	45

P<0.001 (FDR uncorrected), voxels>10

## Discussion

In the present study, we detected the location and degree of cortical activities under the binocular fusion stimulus with fMRI and compared changes between intermittent exotropia and control subjects. Our results showed that the implement of binocular fusion involved with several regions such as middle occipital gyrus, precuneus, fusiform gyrus and so on. For patients with intermittent exotropia, other increased activation intensity areas including bilateral superior parietal lobule and inferior parietal lobule were also observed.

### Fusion functions related brain cortices in normal subjects

Several areas were found to participate in the binocular fusion. Previous animal experiment has reported fusion related activated areas distributed in frontal lobe, posterior parietal cortex, parietal-occipital cortex, midbrain and cerebellum. However, we didn’t find the activation of midbrain and cerebellum in our study. Hasebe et al proved the participation of right fusiform gyrus and left inferior parietal lobule in binocular fusion with PET [16], and Roker reported the activation of middle temporal lobe with the fusion stimulus, both of which are consistent with our results [17].

Both posterior parietal cortex and frontal eye field belong to the spatial attention system [18]. Visual information will be processed by them before transferring to cingulate gyrus. Mesulam believe that the spatial attention network is embedded within an oculomotor network [19]. Human posterior parietal cortex is composed of inferior parietal lobule, superior parietal lobule, and interparietal sulcus. It involves with the dynamic display of object and provides immediate instruction to make attention shift from one object to another. Frontal lobe includes frontal eye field and adjacent parts of premotor area. It may play its critical roles in attentional shifting into specific motor actions. Cingulate gyrus plays a key role in judging the motivational relevance of extrapersonal events and maintaining the level of effort needed for the performance of attentional tasks. All these three cortices provide the local network for regional neural connection on one hand, and also as the nodal points to distribute information on the other hand. Animal experiment proved the above areas’ association with the control or monitor of ocular movement [20–21]. In the present study, the activation of above areas were found with fMRI in normal subjects when performed with fusion, which further reveals the connection between binocular fusion and ocular movement system.

Besides that, Chan et al found the increased volume of caudate nucleus in strabismus with VBM [15]. We also observed the increasing BOLD signal in normal subjects when performing fusion. Caudate nucleus is one part of the dorsal striatum, and is a component of the basal ganglia[22]. Previous study has reported the involvement of caudate nucleus in the motor system to support working memory performance by mediating sensory-motor transformations[23]. Here we speculate caudate nucleus may take charge of connection with other cortices in binocular fusion.

**Fig 4:**
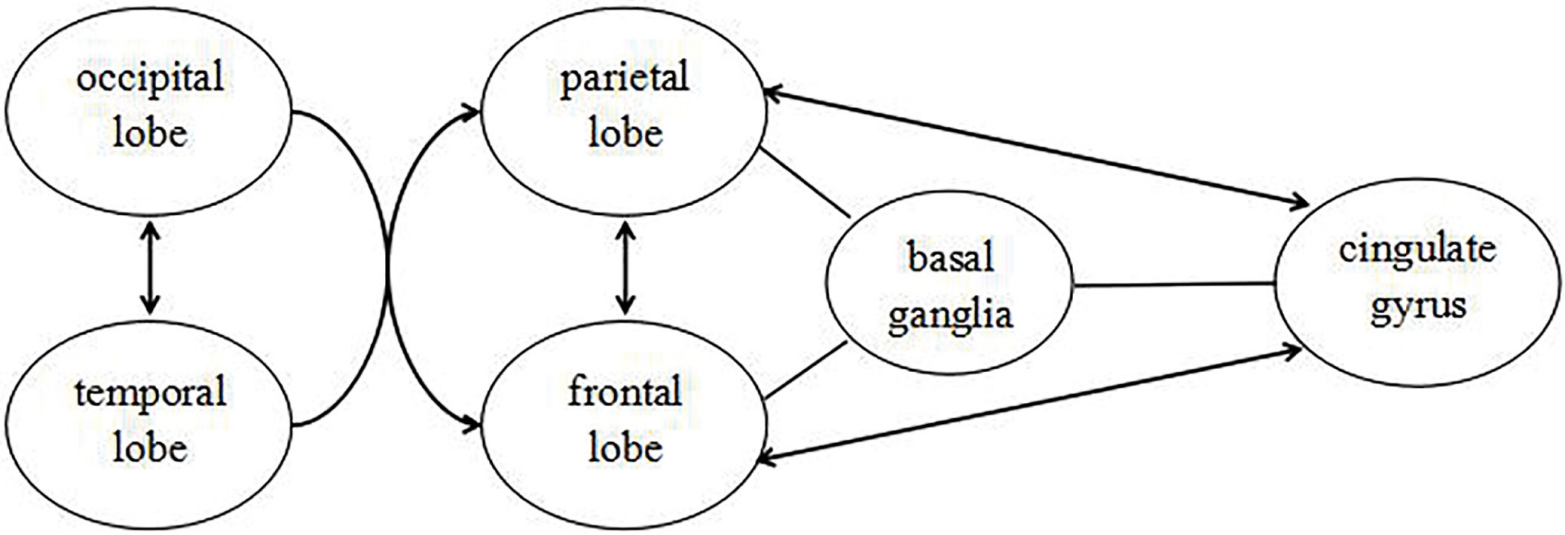
The schematic diagram reflecting the possible pathways of binocular fusion.

Above all, our results indicate that the programming of binocular fusion involves a brain network joined with several regions. Visual information transfers from occipital and temporal lobe to related areas of parietal and frontal lobe, which identify whether fusion function will be needed and how to implement the course of fusion. After that, they connect with cingulate gyrus to monitor and adjust the eye position during the fusion. Besides that, caudate nucleus plays its roles on information processing and connection with other brain regions (Fig 4).

### Fusion functions related brain cortices in intermittent exotropia subjects

Cortical activations in intermittent exotropia were found in bilateral middle occipital gyrus, right inferior temporal lobule, right precuneus and fusiform gyrus and lingual gyrus in right occipital lobe. Middle occipital gyrus, fusiform gyrus and lingual gyrus belong to the part of occipital lobe cortex. As a part of ventral visual pathway, the fusiform gyrus participates in visual perception such as color information processing and face recognition, and it can also project visual information to temporal lobe [24]. Lingual gyrus plays an important role in visual memory [25] and recognition of words [26]. It has been reported the impairment of primary visual cortex with VBM in intermittent exotropia [15]. More activation may be needed to keep the fixation on fusion images during their performance of visual tasks, which is the possible reason why we observed obvious activation in occipital lobe.

Temporal lobe involves with the eye fixation and pursuit movement [27]. Visual disparities of fusion pictures in our study displayed dynamically. Eyes need to adjust fixation and pursuit to keep a single image, which means its more effort for binocular fusion.

We also observed the activation of right precuneus, which indicates that the ocular motor system is not completely impaired and still has some part of activated areas. It coincides well with incomplete binocular fusion loss in intermittent exotropia.

Compared with normal subjects, increased activation intensity of bilateral superior parietal lobule and inferior parietal lobule were found in intermittent exotropia subjects. Both of two regions belong to posterior parietal cortex. Its connection with pre-motor cortex and frontal eye field has been found in animal experiment [28], and some other studies have indicated its connection with spatial attention and fixation [29–30]. In addition, Hasebe et al has reported the participation of posterior parietal cortex in normal control’s binocular fusion [16]. Here we speculate that the increased activation intensity of posterior parietal cortex in our study may reflect a functional compensation for binocular fusion dysfunction in intermittent exotropia.

There are also some limitations. On one hand, considering the cooperation during testing, all subjects we recruited are adults beyond the critical period of vision development. The findings may only reflect the conditions of adult intermittent exotropia. The results of younger intermittent exotropia are still needed to be confirmed in further studies. On the other hand, our study only investigated the cortical activation of intermittent exotropia. As a type of concomitant exotropia, whether our results could be applied to other concomitant exotropia can be further studied.

## Conclusions

1. Binocular fusion involves a complicated brain network including several regions distributed in occipital lobe, temporal lobe, parietal lobe and frontal lobe.
2. Cortical activities of bilateral superior parietal lobule and inferior parietal lobule compensate for the binocular fusion dysfunction in intermittent exotropia.

## Supporting Information

S1 STROBE Checklist (DOCX)
