# Supplementary material for: Assessment of Cortical Dysfunction in Patients with Intermittent Exotropia: An fMRI Study
Source: PLoS One. 2016 Aug 8;11(8):e0160806. doi: 10.1371/journal.pone.0160806 (PMC4976854; doi:10.1371/journal.pone.0160806)
Supplement: S1 STROBE Checklist — (DOCX) [file pone.0160806.s001.docx]

STROBE Statement—checklist of items that should be included in reports of observational studies

|  | Item No. | Recommendation | Page  No. | Relevant text from manuscript |
| --- | --- | --- | --- | --- |
| **Title and abstract** | 1 | (*a*) Indicate the study’s design with a commonly used term in the title or the abstract |  |  |
|  |  | (*b*) Provide in the abstract an informative and balanced summary of what was done and what was found | 2 | In the present study, changes of binocular fusion related cortices in intermittent exotropia were investigated with blood oxygen level dependent functional magnetic resonance imaging. Activated cortices induced by fusion stimulus were showed in some parts of occipital lobe, temporal lobe, parietal lobe and frontal lobe. Compared with normal subjects, increased activation intensity were observed in bilateral superior parietal lobule and inferior parietal lobule in subjects with intermittent exotropia. |
| Introduction | | | |  |
| Background/rationale | 2 | Explain the scientific background and rationale for the investigation being reported | 2-4 | The pathological mechanisms of intermittent exotropia might be associated with multiple factors. The defective binocular fusion was more considered as the possible cause of intermittent exotropia.  Previous studies about intermittent exotropia mainly concentrated on its clinical characteristics and the outcomes of operation. Neural mechanisms are rarely mentioned. Connections between brain cortex and strabismus have been reported in some studies. Characteristics of strabismus may vary greatly in different types. As a special type of concomitant exotropia, the recognition of neural mechanisms in intermittent exotropia may help us to have a deep understanding about strabismus and provide theory evidences for treatment. |
| Objectives | 3 | State specific objectives, including any prespecified hypotheses | 4 | In the present study, a series of pictures with visual disparities were used to produce binocular fusion visual stimulus. We explored the binocular fusion related cortices in intermittent exotropia and normal subjects with blood oxygenation level dependent-functional magnetic resonance imaging (BOLD-fMRI). It will be helpful for us to further understand the binocular fusion of intermittent exotropia and the neural mechanisms of strabismus. |
| Methods | | | |  |
| Study design | 4 | Present key elements of study design early in the paper | 4 | In this cross-sectional study, nine intermittent exotropia subjects and eight control subjects were recruited in West China Hospital of Sichuan University from July 2015 to February 2016. |
| Setting | 5 | Describe the setting, locations, and relevant dates, including periods of recruitment, exposure, follow-up, and data collection | 4 | nine intermittent exotropia subjects and eight control subjects were recruited in West China Hospital of Sichuan University from July 2015 to February 2016. |
| Participants | 6 | (*a*) *Cohort study*—Give the eligibility criteria, and the sources and methods of selection of participants. Describe methods of follow-up  *Case-control study*—Give the eligibility criteria, and the sources and methods of case ascertainment and control selection. Give the rationale for the choice of cases and controls  *Cross-sectional study*—Give the eligibility criteria, and the sources and methods of selection of participants | 4-5 | All subjects were right-hand and had no history of amblyopia and other ocular diseases, psychiatric disorders or brain abnormalities. |
|  |  | (*b*)*Cohort study*—For matched studies, give matching criteria and number of exposed and unexposed  *Case-control study*—For matched studies, give matching criteria and the number of controls per case |  |  |
| Variables | 7 | Clearly define all outcomes, exposures, predictors, potential confounders, and effect modifiers. Give diagnostic criteria, if applicable | 7 | significant activated areas were defined as the number of continuous activated voxels reached more than 10 voxels. |
| Data sources/ measurement | 8* | For each variable of interest, give sources of data and details of methods of assessment (measurement). Describe comparability of assessment methods if there is more than one group | *5-6* | *MRI scannings were performed in a 3.0T MR scanner system with a standard eight-channel head coil (EXCITE, GE Signa, Milwaukee, USA). Three dimensional T1 weighted anatomical images were collected with the following scan parameters: repetition time (TR) =8.5ms, echo time (TE) =3.4ms, flip angle=12°, slice thickness=1mm. Functional images were obtained with an echo planar imaging (EPI) sequence, and the scan parameters were as follows: TR=2000ms, TE=30ms, flip angle=90°, matrix=64×64, field of view=240×240mm2, slice thickness=5mm.* |
| Bias | 9 | Describe any efforts to address potential sources of bias | 6 | During the scanning, foam pads were filled around head and earplugs for all subjects to minimize head motion and scanner noise, respectively.  The first twenty volumes were discarded to ensure stable magnetization and subjects’ adaption to the circumstances. Slice timing and realignment for head motion correction were then performed for the remaining images.  Data of one intermittent exotropia subject and one control subject were excluded from following analysis because either translation or rotation of their head motion exceeded 1mm or 1° on any axis. |
| Study size | 10 | Explain how the study size was arrived at | 6 | Data of one intermittent exotropia subject and one control subject were excluded from following analysis because either translation or rotation of their head motion exceeded 1mm or 1° on any axis. |

Continued on next page

| Quantitative variables | 11 | Explain how quantitative variables were handled in the analyses. If applicable, describe which groupings were chosen and why |  |  |
| --- | --- | --- | --- | --- |
| Statistical methods | 12 | (*a*) Describe all statistical methods, including those used to control for confounding | 7 | For fMRI data, after preprocessing in SPM8, data from subjects in the same group were combined and statistically analyzed to obtain the averaged images of activation. The paired t-test was applied to compare the differences of activated areas induced by fusion stimulus and control stimulus in the same group. And the group t-test was used to compare the differences induced by stimulus. A false discovery rate (FDR) was set at a level of P<0.001, uncorrected, and the extent threshold was set with 10 voxels.  For demographic comparisons, the statistical analysis was performed with SPSS Statistics (SPSS Inc, Version 18.0, Chicago, USA). Two-sample two-tailed t-test was performed for age comparison and Fisher’s exact probability test for gender. Significant difference was set at a level of p<0.05. |
|  |  | (*b*) Describe any methods used to examine subgroups and interactions |  | There is not any subgroup in our study. |
|  |  | (*c*) Explain how missing data were addressed | 6 | Data of one intermittent exotropia subject and one control subject were excluded from following analysis. |
|  |  | (*d*) *Cohort study*—If applicable, explain how loss to follow-up was addressed  *Case-control study*—If applicable, explain how matching of cases and controls was addressed  *Cross-sectional study*—If applicable, describe analytical methods taking account of sampling strategy |  |  |
|  |  | (*e*) Describe any sensitivity analyses |  |  |
| Results | | | | |
| Participants | 13* | (a) Report numbers of individuals at each stage of study—eg numbers potentially eligible, examined for eligibility, confirmed eligible, included in the study, completing follow-up, and analysed |  |  |
|  |  | (b) Give reasons for non-participation at each stage |  |  |
|  |  | (c) Consider use of a flow diagram |  |  |
| Descriptive data | 14* | (a) Give characteristics of study participants (eg demographic, clinical, social) and information on exposures and potential confounders | 7 | Demographic and clinical information of 8 intermittent exotropia subjects (3 males, 5 females; age: 23.8±6.8 years) were displayed in Table 1. In addition, 7 normal control subjects (3 males, 4 females; age: 25.0±2.1 years) were well matched in age (two-sample two-tailed t-test, t = -0.443, p = 0.669) and gender (Fisher’s exact probability test, p>0.99). |
|  |  | (b) Indicate number of participants with missing data for each variable of interest |  |  |
|  |  | (c) *Cohort study*—Summarise follow-up time (eg, average and total amount) |  |  |
| Outcome data | 15* | *Cohort study*—Report numbers of outcome events or summary measures over time |  |  |
|  |  | *Case-control study—*Report numbers in each exposure category, or summary measures of exposure |  |  |
|  |  | *Cross-sectional study—*Report numbers of outcome events or summary measures |  |  |
| Main results | 16 | (*a*) Give unadjusted estimates and, if applicable, confounder-adjusted estimates and their precision (eg, 95% confidence interval). Make clear which confounders were adjusted for and why they were included |  |  |
|  |  | (*b*) Report category boundaries when continuous variables were categorized |  |  |
|  |  | (*c*) If relevant, consider translating estimates of relative risk into absolute risk for a meaningful time period |  |  |

Continued on next page

| Other analyses | 17 | Report other analyses done—eg analyses of subgroups and interactions, and sensitivity analyses |  |  |
| --- | --- | --- | --- | --- |
| Discussion | | | | |
| Key results | 18 | Summarise key results with reference to study objectives | 10-11 | In the present study, we detected the location and degree of cortical activities under the binocular fusion stimulus with fMRI and compared changes between intermittent exotropia and control subjects. Our results showed that the implement of binocular fusion involved with several regions such as occipital lobe, temporal lobe, parietal lobe and frontal lobe. For intermittent exotropia subjects, other increased activation intensity areas including bilateral superior parietal lobule and inferior parietal lobule were also observed. |
| Limitations | 19 | Discuss limitations of the study, taking into account sources of potential bias or imprecision. Discuss both direction and magnitude of any potential bias | 14 | There are also some limitations. On one hand, considering the cooperation during testing, all subjects we recruited are adults beyond the critical period of vision development. The findings may only reflect the conditions of adult intermittent exotropia. The results of younger intermittent exotropia are still needed to be confirmed in further studies. On the other hand, our study only investigated the cortical activation of intermittent exotropia. As a type of concomitant exotropia, whether our results could be applied to other concomitant exotropia can be further studied. |
| Interpretation | 20 | Give a cautious overall interpretation of results considering objectives, limitations, multiplicity of analyses, results from similar studies, and other relevant evidence |  |  |
| Generalisability | 21 | Discuss the generalisability (external validity) of the study results | 14 | The findings may only reflect the conditions of adult intermittent exotropia. The results of younger intermittent exotropia are still needed to be confirmed in further studies.  On the other hand, our study only investigated the cortical activation of intermittent exotropia. As a type of concomitant exotropia, whether our results could be applied to other concomitant exotropia can be further studied. |
| Other information | |  | | |
| Funding | 22 | Give the source of funding and the role of the funders for the present study and, if applicable, for the original study on which the present article is based |  |  |

*Give information separately for cases and controls in case-control studies and, if applicable, for exposed and unexposed groups in cohort and cross-sectional studies.

**Note:** An Explanation and Elaboration article discusses each checklist item and gives methodological background and published examples of transparent reporting. The STROBE checklist is best used in conjunction with this article (freely available on the Web sites of PLoS Medicine at http://www.plosmedicine.org/, Annals of Internal Medicine at http://www.annals.org/, and Epidemiology at http://www.epidem.com/). Information on the STROBE Initiative is available at www.strobe-statement.org.
